# Polydrug Definition and Assessment: The State of the Art

**DOI:** 10.3390/ijerph192013542

**Published:** 2022-10-19

**Authors:** Sílvia Font-Mayolas, Fran Calvo

**Affiliations:** Quality of Life Research Institute, Universitat de Girona, Plaça Sant Domènec, núm. 9, 17004 Girona, Catalonia, Spain

**Keywords:** polydrug use, definition, assessment, evaluation, state of the art, review

## Abstract

Polydrug use is a very common phenomenon and represents an important public health problem. The definition of the term has varied since its inception, and consequently so have forms of self-report evaluation. The aim of this review is to offer an overview of how the concept has evolved and its forms of evaluation through self-reporting. A search of the term polydrug was conducted on the PubMed portal up to August 2022, with a total of 2076 publications detected containing the word polydrug in their title, abstract or keywords. This includes publications that represent an advance in the definition and assessment of this construct through self-reports, which may be useful for researchers carrying out future studies in the field. The importance of distinguishing between concurrent and simultaneous polydrug use and the need to employ comparable measures in parameters for the frequency, magnitude and combination of psychoactive substances involved in polydrug use are two of the main recommendations emerging from this review.

## 1. Introduction

Studies on the use of psychoactive substances have often been conducted by means of substance-by-substance analysis, meaning it is common to find research aimed at determining how many people smoke tobacco or consume kratom and the characteristics of such consumption. However, this fails to address the issue of which is more frequent: the consumption of one single psychoactive substance or of more than one at the same time. Studies on the prevalence of consumption show that it is more of a rule than an exception that the same person may be a user of more than one psychoactive substance, with the worrying associated increase in health risks [1].

If we consider types of treatment request when it comes to drug use, according to Montanari and Guarita [2], a large proportion of drug treatment clients in Europe consume more than one drug, the most common combinations being the following: opioids, cannabis and cocaine; cocaine, cannabis and alcohol; and a stimulant, alcohol and cannabis. In addition, the European Monitoring Centre for Drugs and Drug Addiction (EMCDDA) [3] has identified three profiles among treatment applicants: co-users of heroin and cocaine; co-users of cocaine, cannabis and alcohol; and co-users of cannabis and alcohol. Alcohol is the drug most present in more than one polydrug use repertoires. Hence, the importance of recognizing polydrug use [4,5].

The definition of polydrug use has varied since its inception, and consequently, so have forms of self-report evaluation. Currently, the World Health Organization (WHO) [6] understands the term polydrug use as the consumption of more than one kind of drug by an individual. According to the EMCDDA [4], the term encompasses the use of both illicit drugs and legal substances, such as alcohol and medicines, either at the same time or sequentially.

An increased tendency in the evaluation of this phenomenon has provided more complete data at both the individual level and the level of global consumer trends. The aim of this review is to offer an overview of how the concept of polydrug has evolved and its forms of evaluation through self-reporting, since this may prove useful in the approach adopted to the phenomenon in future studies.

## 2. Materials and Methods

This state-of-the-art review takes into account publications that contain the keyword “polydrug” in their title, abstract or keywords published on the PubMed portal from its inception until August 2022. There were 2076 results in total. A chronological review of the publications was carried out in order to offer an evolutionary analysis of the concept and its forms of evaluation. After reviewing all of them, this article includes those that, in the opinion of the authors, represent an advance in the definition and evaluation of the concept.

## 3. Results

### 3.1. The Beginnings

**Figure 1:**
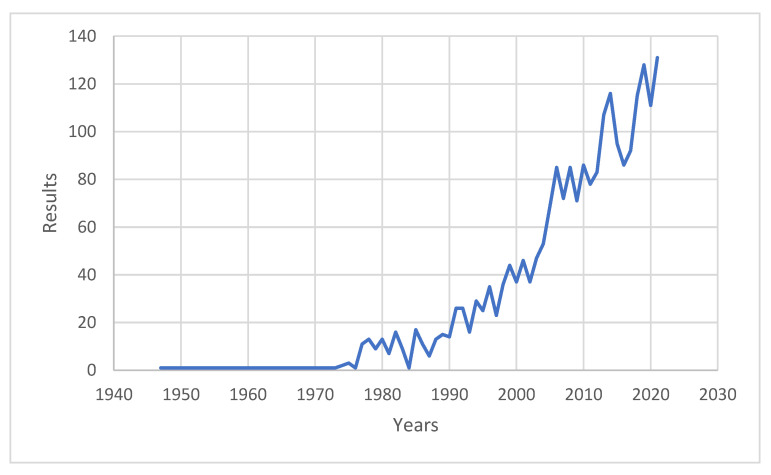
Results of search for articles with the word polydrug use in title, abstract or keywords in PubMed up to August 2022.

The first publication to include the word “polydrug use” on the PubMed portal dates back to 1947; however, in this instance it was applied in the medical field of polyresistances to medicines. It was not until 1973 that a second work appeared with this term—written as “poly-drug”—though still closely linked to drug combinations [7]. Then, in 1974 two works [8,9] appeared in which the concept of polydrug was clearly situated in the field of addictions (Figure 1).

The annual number of articles citing the term increases from the 1980s onwards, from around 10 to 25 per year in the nineties, then progressively rises to the 128 works published in 2019, 114 in 2020 and 131 in 2021. A review of these works allows us to observe that the social sciences have progressively been incorporated into the study of this topic initially analysed from the perspective of the natural sciences.

### 3.2. Initial Definitions

If we delve further into the meaning of the term “polydrug use”, we find an early operational definition in a 1975 book by the National Institute on Drug Abuse (NIDA). This was the first work that attempted to find a unique definition for the concept “polydrug use” based on initial works carried out in this field. It is here that we encounter the first difficulties in defining the concept, as, in the words of Johnston [10], it took on three possible labels: (1) “Ever use”, or past use of multiple drugs; (2) Recent use of multiple drugs; and (3) Overlapping use of multiple drugs: for enhanced effects, for offsetting or smoothing effects, or for indeterminate effects. Of these, Johnston [10] highlighted the first, since he considered that it might include the others, although he did recommend specifying which label was being used in each future investigation. The same work addressed some conceptual issues surrounding this concept, with the proposals that drugs considered legal (alcohol, tobacco and caffeine) be included within the term polydrug, and anyone using two or more drugs of the same type, different types of amphetamines, for example, not be considered polyusers.

In a later work by Kaufman [11], a distinction was made between the concept of “polydrug abuse”, understood as dependence on psychoactive substances in which the primary dependence is not heroin, cocaine or alcohol, and “multidrug abuse”, understood as substance abuse with a different primary dependence, Kaufman advocating the use of the latter term. As for evaluation of this behaviour, given that the total possible combinations of psychoactive substances would be in the billions, an initial proposal was made to classify people who consume more than one drug into four groups: narcotic abuse (with the abuse of other psychoactive drugs and alcohol); methadone maintenance (with the abuse of other drugs and alcohol); alcohol abuse (with the abuse of other drugs); and abuse of more than one non-narcotic drug.

A year later, in a paper entitled “Polydrug abuse or multidrug misuse: it’s here to stay”, Kaufman [12] considered that the concept “polydrug” may be confused due to the definition awarded to it by the NIDA in applications for research grants (dependence on a psychoactive drug or drugs in which the primary dependence was not heroin, methadone or alcohol). To clarify the definition, then, Kaufman [12] returned to his four consumption groups and reserved the term “polydrug” for only the fourth group: primary abuse of more than one psychoactive drug (without heroin or alcohol dependence).

The above being said, repeating the search on the PubMed portal but now using the term “multidrug abuse” up to August 2022 returned a total of 482 results. The word multidrug appeared linked to the consumption of drugs in scientific publications mainly in the 1970s, 1980s and 1990s, before its appearance progressively decreased. Simultaneously, the use of the term multidrug has been found in the medical field, specifically in relation to resistance in bacteria, corresponding to approximately 80% of the entries for this term.

From 1984 onwards, the term “polydrug use” appears as a synonym for “polysubtance use”, with the result that we find works that opt for this synonym in this topic area and works in which the use of both terms is combined in the same publication.

### 3.3. Development of the Concept and Its First Forms of Evaluation

In terms of evaluation, the first two labels proposed in Johnston’s initial definition [10] can easily be evaluated using a list of psychoactive substances and counting those for which the person gives an affirmative answer. However, in the case of the third label, evaluation is complicated by the large number of possible combinations of drugs. This difficulty, according to Johnston [10], could be overcome by choosing only those substances of greatest interest on the part of the researcher, by combining substances only in twos, or by asking for combinations only when the person has already reported use of one of the substances.

One of the first measures we found was from 1978, when Douglass and Khavari [13] proposed a quantitative index to assess degree of involvement in polydrug use. They called this measure the DUI (Drug Use Index). To this end, they used the scale to record the consumption of 19 types of drugs: not at all; less often than monthly; about once a month; about once a week; daily; and several times a day. If a person’s DUI value exceeds the population average by four typical deviations, then that person’s involvement in drug use is considered to be significantly higher than that of their reference population. In the same year [14], in the absence of psychometric instruments to evaluate polydrug drug use, the same authors created the SBP (Polydrug Assessment Scale), an indirect scale comprising 32 items similar to the items found in personality tests (for example: “I have participated in a political demonstration” or “I have few or no pains”), with which they obtained a stable correlation with polydrug use.

Morrisey [15] also addressed measuring polydrug use through four different indices: (1) the number of substances consumed; (2) the establishment of weights per substance based on their consumption in the population, from which to establish an average score for each subject; (3) a scale of involvement (which includes severity of the effects of each substance, frequency of use, method of administration, duration of use and age of first use); and (4) a hierarchical management of drug use from alcohol to opiates. When the four forms of measurement are compared, a high correlation is detected between the first three indices and, to a lesser extent, between these and the fourth. The difficulty of quantifying lifetime use of the different psychoactive substances is noted, taking into account the possible differences in frequency and duration of each.

Subsequent works observed how measuring polydrug use based on the sum of the substances consumed in different periods of time has been maintained until today. Thus, for example, an article by Martin et al. [16] presented data grouped by number of psychoactive substances consumed in North American adolescents and proposed patterns based on combinations of substances. In the same line, Boys et al. [17] examined polydrug patterns at a rave party in Australia, also from the sum of the psychoactive substances used. Furthermore, Schuler et al. [18] used the sum of psychoactive substances consumed and distinguished between levels of polydrug use: two substances and three or more substances. Other authors who have used summation as a way to evaluate polydrug have considered it only from the consumption of three or more substances [19,20].

A new contribution to polydrug use assessment came with the work by Sneed et al. [21], in which evaluation is proposed through three indices: (1) the number of substances consumed; (2) weighting consumption by awarding weights according to the severity of the substance consumed (cigarettes = 1, alcohol = 2, etc.); and (3) a hierarchy based on Kandel and Yamagutchi’s gateway theory [22] of awarding weights according to progress (cigarettes or alcohol = 1, cigarettes and alcohol = 2, etc.). The severity of consumption can be gauged using Indexes 2 or 3.

In 2009, the EMCDDA [3] proposed the evaluation of three types of polydrug use by grouping the substances consumed: alcohol and tobacco (type A); cannabis together with alcohol and/or tobacco (type B); and cannabis together with alcohol and/or tobacco and at least one of the following substances: ecstasy, cocaine, amphetamines, LSD or heroin (type C). This evaluation determines the most frequent type of polydrug among those proposed according to the study population [23,24].

A further step in polydrug assessment came with the application of latent class analysis to the data obtained. Thus, Sañudo et al. [25] asked about frequency of consumption of a wide list of psychoactive substances over the past year and binge drinking in the case of alcohol, obtaining the following three groups: (1) no polydrug use; (2) moderate polydrug use (high probability of smoking tobacco, moderate probability of binge drinking and cannabis use, and low probability of use of other drugs); and (3) high-level polydrug use (high risk of binge drinking and use of cannabis and ecstasy, and moderate probability of consumption of other drugs).

Martínez-Loredo et al. [26] also employed the analysis of latent classes together with consumption frequency of each substance over the past year in polydrug evaluation (in their case only for tobacco, alcohol and cannabis). They identified three trajectories in polydrug use: (1) early onset (tobacco and cannabis users); (2) experimental use (no substance use together with those presenting a stable pattern of moderate use of alcohol in absence of other drug use); and (3) telescoped use (no frequent use of any substance).

This way of evaluating polydrug use by means of latent class analysis and taking into account parameters such as frequency of consumption and how substances are combined has been further developed up until today [27,28,29] and is considered useful for the detection of high-risk groups when applying selective prevention [30].

### 3.4. Incorporation of Simultaneity in Polydrug Use Assessment

A turning point came with the realization of the need to distinguish between concurrent and simultaneous polydrug use. In 1990, Grant and Harford [31] analysed concurrent and simultaneous polydrug use as part of the National Survey on Drugs in the United States. Concurrent polydrug use is understood as the consumption of alcohol and other substances during the same time period, namely over the previous year. Simultaneous polydrug use, specifically alcohol together with sedatives or tranquilizers, refers to the use of alcohol together with these substances at the same time (or in an interval of two hours) during the last year. A further work by the same authors from the same year analysed concurrent and simultaneous polydrug use, in this case of alcohol and cocaine, in the last month and in the last year [32].

In the same vein, Martin et al. [33] examined concurrent and simultaneous polydrug use in English university students. The authors asked about the use of the following substances: alcohol, tobacco, cannabis and hallucinogens. To assess concurrency, participants were asked about “at the same time” use. More specifically, the instructions included the following explanation: “By ‘at the same time’, we mean using another drug while you are still using or feeling the effects of the first drug”.

Earleywine and Newcomb [34] also highlighted the need to distinguish between concurrent (several drugs used on separate occasions) and simultaneous (the use of more than one drug at the same time) polydrug use. To evaluate the former, the authors asked about frequency of consumption over the previous six months of: cigarettes, alcohol, cannabis and other illegal drugs (cocaine, hypnotics, stimulants, narcotics, PCP, inhalants and hallucinogens), using a scale ranging from “No consumption (0)” to “More than once a day (7)”. For simultaneous polydrug assessment, participants were asked to answer the number of times they had consumed the following pairs of substances at the same time over the previous six months: cigarettes and alcohol; cigarettes and cannabis; cigarettes and other illegal drugs; alcohol and cannabis; alcohol and other illegal drugs; and cannabis and other illegal drugs. The authors created these groupings from the review of the most consumed substances in previous epidemiological studies. For these authors, the different prevalence detected for the two types of polydrug use (concurrent and simultaneous) shows that they clearly represent two different phenomena. That is, there are people who report having used two psychoactive substances in the last six months but never at the same time, for example. The authors detected a high correlation between both types of polydrug use, while at the same time observing a high discriminant validity for each of these constructs.

Collins et al. [35] then published a paper on simultaneous polydrug use in American adolescents. For evaluation, participants were asked how many times they had consumed the following pairs of substances on the same occasion in the past year: alcohol with downers (depressants); alcohol with uppers (stimulants); alcohol with marijuana; cocaine or crack, along with another drug. A scale of 0 (on no occasion) to 6 (40 times or more) was used. The use of this type of measure allowed the authors to delve into the most common drug combinations in polydrug use, with the simultaneous combination of alcohol and marijuana being the most frequent. These authors also identified the difficulty of delimiting the wide number of possible combinations of drugs.

McCabe et al. [36] also evaluated concurrent and simultaneous polydrug use in American university students in relation to alcohol and non-prescription drugs. To assess polydrug use, they asked: “In the past 12 months, how many days have you used prescription stimulant medication (e.g., methylphenidate) not prescribed to you by a doctor at the same time you were drinking alcohol?” The same question was asked for simultaneous use of alcohol and for each of the following drug classes: pain medication; sedative/anxiety medication; and sleeping medication. Concurrent polydrug use was evaluated by means of the same question, but with the nuance “but no simultaneous polydrug use”. These authors stressed the importance of continuing to collect data on polydrug use by distinguishing between these two forms.

Martin’s work [37] also highlighted the possibility of classifying types of polydrug use according to the time that the various substances were ingested. Thus, they described concurrent polydrug use as the use of two or more substances over a period of time, such as a year or a month. Simultaneous polydrug use was understood as the use of two or more substances in combination (for example: at the same time or with temporal proximity). The author pointed out that all simultaneous polyusers are concurrent polyusers, while concurrent polyusers may or may not be simultaneous polyusers. Likewise, the author also pointed out that most simultaneous polyusers show a high intentionality when choosing combinations of substances to obtain the expected effects. A motivation may be additive or interactive effects (as in the combination of alcohol and cocaine) or reduction or mitigation effects (as in the combination of cocaine and sedatives), which opened up a new field of study in polydrug use. Proof of this is the review by Bolieau-Falardeau et al. [38], in which self-medication for a pre-existing condition is referred to as a possible third motivation, and the review by King & Meyer [39], studying the role of nicotine as a stimulant for the abuse of drugs such as alcohol.

Quek et al. [40] also analysed concurrent and simultaneous polydrug use, specifically in a population of young Australian adults. To assess concurrent polydrug use, they asked about the consumption of a wide list of drugs over the previous 12 months. To assess simultaneous polydrug use, the question was: “Which of the following did you use at the same time, on at least one occasion that you used (drug type)? With a list of possible drugs”. These authors calculated the percentage of coincidence between both types of polydrug use based on the combination of substances. Furthermore, they applied an analysis of latent classes to detect types of consumption.

Baggio et al. [41,42] analysed concurrent and simultaneous polydrug use in a sample of young Swiss men. Concurrent polydrug use was assessed on the basis of use/non-use of drugs (alcohol, tobacco and 15 other substances) over the past year. A concurrent polydrug use index was obtained from the sum of all categories of drugs consumed over the past 12 months. Simultaneous polydrug use was assessed by asking participants for the usual number of drugs consumed on the same occasion and the maximum number of drugs combined, obtaining a usual simultaneous polydrug global index and a maximum simultaneous polydrug index. In line with the findings of the previous works, the authors concluded that although concurrent and simultaneous polydrug use are related, they are two different constructs.

With regard to measurement, it is worth noting the work that Karjalainen et al. [43] dedicated to forms of evaluation for concurrent polydrug use from the analysis of a sample of the general Finnish population. These authors proposed four categories for assessing concurrent polydrug use (two psychoactive substances): strict (last 30 days, in the case of alcohol must be binge drinking, understood as between at least 6–7 units of alcohol per occasion, tobacco is not included); medial (a) (past 12 months, in the case of alcohol must be binge drinking, tobacco is not included); medial (b) (past 12 months, including any amount of alcohol, tobacco is not included); and loose (past 12 months, including any amount of alcohol use and current daily smoking). These authors detected that polydrug use varies according to the measure used. Thus, asking about the past 12 months can overestimate polydrug use if compared to asking about the past month, especially if frequency and magnitude of consumption are not taken into account. These authors also noted that the type of substances included in the unit of measurement also influences prevalence trends. Since it is difficult to take preventive measures in health if the measures are not refined, the authors pointed to the need to evaluate simultaneous polydrug use as the best way of understanding the phenomenon of polydrug use.

Garrido et al. [44] evaluated simultaneous polydrug use in Spanish university students, without further specifying the format of the question, and focused on the past 30 days. Additionally, with Spanish university students, Hernández-Serrano et al. [45] evaluated both concurrent and simultaneous polydrug use; more specifically, they asked when, over the past 12 months, the person had smoked: cannabis and tobacco separately; cannabis mixed with tobacco in joints; and cannabis mixed with tobacco in joints together with alcohol.

### 3.5. Other Developments in Polydrug Use Assessment

Interest in polydrug use has led to the coining of specific terminology in the case of nicotine consumption, with researchers employing the term “polytobacco use”. A search for this word on the PubMed portal resulted in 171 publications, the numbers again increasing over the years. The first publication dates from 2007, when the term polytobacco is described as the concurrent use of cigarettes and one or more other tobacco products [46]. Other authors have used the term “dual” to refer to the consumption of two tobacco products and reserve the word polytobacco for the consumption of three or more [47]. As with polydrug use, when evaluating polytobacco use, the combinations of substances taken into account, the number of them, and frequency and magnitude all vary [48,49]. Consequently, the challenges in improving its evaluation are similar to those mentioned for the assessment of polydrug use.

Finally, the interest in polydrug use has translated into the use of new forms of assessment beyond self-reports, blood analyses [50] and urine tests [51], such as analysing the content of used syringes [52], the toxicological analysis of post-mortem specimens [53], hair analysis [54], evaluation of latent fingerprints [55], oral fluid analysis [56], social media data analysis [57], the literal test analysis of polydrug overdose death (from death certificate data) [58] and trace residue sampling strategies (analysis of discarded drug packaging samples) [59].

## 4. Conclusions

This review regarding the definition and measurement of polydrug use contributes a selection of data that may be key for future research (Table 1).

Thus, some conclusions can be proposed based on the review. In line with that pointed out by Kataja et al. [60], although many of the published works on polydrug consumption come from the field of natural sciences (toxicology, pharmacy or neurosciences), it is observed that, as happened previously with individual substance use, polydrug use is also progressively being analysed and evaluated employing theories from the social sciences.

The growing volume of publications on polydrug use implies that this phenomenon is increasingly being taken into account in studies on the prevalence of drug use, and that its study should therefore be incorporated into such surveys, especially of a national or continental nature, in order to offer a more concrete overview of consumption. Knowing to what extent drug users use more than one substance, what types of substances are combined and their effects, can be very useful at the national government level to establish prevention strategies in this regard.

Publications continue to be detected in which the term polydrug is used directly without being accompanied by its definition, meaning interpretation of what is meant by polydrug use is in the hands of the reader. In future works, it is recommended that researchers wishing to collect data on polydrug use employ an operational definition and include it in their publications.

The full picture of the polydrug use phenomenon involves assessment of concurrent and simultaneous polyuse. Taking concurrence and simultaneity into account can facilitate a form of treatment at the clinical level that is even more adapted to the needs of those polydrug users at the idiographic level; that is, an intervention that considers the effects of specific combinations in order to facilitate the process towards abstinence. In the words of the EMCDDA [4], not taking this information into account is losing an opportunity to increase the probability of achieving the therapeutic objectives.

In future research on both concurrent and simultaneous polydrug use, depending on aims attention should be paid to the period of time referred to—the most common in the literature being the past 30 days and the past 12 months—in order to guarantee comparability of results. The frequency and magnitude of consumption of each of the psychoactive substances used should also be clarified, taking into account the different reinforcing effects of each substance, which may imply divergences between frequency and preference, the most consumed substance not always being the most preferred [61].

In the case of simultaneous polydrug use, in future works it will be necessary to bear in mind how to word the possible combinations of substances being studied. Furthermore, future research will need to take into account the very large number of psychoactive substances in existence and prioritize which to include in accordance with the aims established. Likewise, the order of combination must also be taken into account, since this may correspond to different intentions on the part of the consumer. Furthermore, the specific time interval recorded with regard to combinations must be included in the question, since this period has been found to vary in the previous literature. In addition to all of the above, the questions should not be of excessive complexity, and be understandable and not tiring for respondents.

Given the various different ways of measuring polydrug use by self-reporting, standardizing the measures would help advance and expand our knowledge of this phenomenon.

## Figures and Tables

**Table 1:** Main information to take into account in research on polydrug use.

Polydrug Use	Key Information
Definition	The term “polydrug” (or polysubstance use) has imposed itself over the term “multidrug”, which was situated mainly in the medical field of resistance in bacteria.
	Currently, polydrug use is defined as the use of more than one kind of substance (whether illicit or legal) by an individual, either at the same time or sequentially.
	The EMCDDA [4] proposes a classification of polydrug use types according to substance combination: Type A (alcohol and tobacco); Type B (cannabis together with alcohol and/or tobacco); and Type C (cannabis together with alcohol and/or tobacco and at least one of the following substances: ecstasy, cocaine, amphetamines, LSD or heroin).
Assessment	The assessment of polydrug use began with the incorporation of measures aimed at defining severity of consumption by awarding weights according to type of substance or combinations thereof, and the frequency and duration of consumption of each substance or combination.
	Analysing simultaneity of use allows for a distinction to be made between concurrent and simultaneous polydrug use, these being two constructs that correlate but are distinguished at the level of discriminant validity (different intentionality of consumption, for example).
	Combinations of substances, the period of consumption being referred to and the types of scales used to evaluate frequency of consumption all vary in concurrent and simultaneous polydrug use assessment. The main difficulties arise from delimiting the large number of combinations of psychoactive substances and, in the case of simultaneous consumption, the time intervals recorded.
